# Health risk assessment of instant noodles commonly consumed in Port Harcourt, Nigeria

**DOI:** 10.1007/s11356-017-0583-0

**Published:** 2017-11-11

**Authors:** Iniobong A. Charles, Atieme J. Ogbolosingha, Inimfon U. Afia

**Affiliations:** 1 Department of Oncology, School of Medicine,Faculty of Medicine and Health Sciences,, City Hospital Campus, University of Nottingham, NG5 1PB, Nottingham, United Kingdom; 2 Department of Biochemistry, Faculty of Science, Federal University Otuoke, PMB 126, Otuoke, Nigeria; 3 Department of Biochemistry, University of Port Harcourt, PMB 5323, Choba, Nigeria

**Keywords:** Instant noodles, Heavy metals, PAHs, Health risk, Carcinogenic risk

## Abstract

The current study investigated the levels of some heavy metals [lead (Pb), arsenic (As), nickel (Ni), mercury (Hg), copper (Cu), cadmium (Cd), aluminum (Al), and chromium (Cr)] and polycyclic aromatic hydrocarbons (PAHs) in six brands of instant noodles (CFN, GFC, NGP, GAA, CUN, and FCS) commonly consumed in Port Harcourt, Nigeria. Risks of consumption of contaminated noodles were also assessed. Heavy metal content and PAHs were determined using flame atomic absorption spectrophotometer and gas chromatography, respectively. Concentrations of heavy metals as Pb, Ni, Cu, Al, and Cr were detected while As, Hg, and Cd were not detected in noodles. High average concentrations (mean ± SD mg/kg) of Pb were observed in brands CFN (3.163 ± 0.21) and GFC (1.022 ± 0.08) which were significantly higher (*P* ≤ 0.05) than in NGP (0.043 ± 0.15) and GAA (0.276 ± 0.18), although all were above WHO permissible limits (0.025 mg/kg). Target Hazard Quotient and Hazard Index for Pb were > 1 in brands CFN and GFC indicating unacceptable risk. Results of PAHs showed brands had total PAHs (mg/kg) in the order CFN > CUN > GAA > NGP > FCS > GFC. Although carcinogenic risks associated with these noodles are within permissible range, consumption of CFN and GFC could pose greater health risk to consumers. Long-term consumption of brands CUN, CFN, and GAA may have higher probability of carcinogenesis among consumers. We therefore recommend more diligent regulatory policies and monitoring by relevant government agencies (WHO, NAFDAC, CPC, and SON) to ensure wholesome noodles get to consumers.

## Introduction

Food is one of the basic needs of man and nature has provided abundant resources for man’s exploitation and use. Interestingly, technological advancement in the food industry in the last five decades has resulted in the production of ready meals and convenience food, thus ensuring the availability of food for immediate consumption. Instant noodles is one of the most common convenience foods in Nigeria, with a high category penetration of about 60% and market size of about 250,000 t in 2012 (Tolaram Group 2012). With just four (4) brands in 2006, the Nigeria instant noodles market now boasts of up to 16 competing brands (Marketing Edge 2017).

Instant noodle is consumed on a global scale and is second only to bread (World Instant Noodles Association 2016). As the consumption of instant noodles continues to grow globally, especially among children, the processes involved in the production and packaging of these noodles become a global concern.

The occurrence of foreign materials in food has always elicited strong response from governments, and unraveling the possible occurrence of toxic substances in food continues to draw the attention of researchers. The use of contaminated raw materials has been identified as a major source of food contamination (Sousa 2008). Heavy metals such as cadmium, lead, mercury, arsenic, aluminum, and chromium are toxic even in minute concentrations (Das 2010). Moreover, these metals have the capacity to bioaccumulate and thus, could trigger irreversible deleterious effects on humans that consume such contaminated food (Jarup 2003). This may include serious threats like renal failure, liver damage, neurological impairment, cardiovascular diseases, and even death (Al-Busaidi et al. 2011).

Lead (Pb) has no known beneficial function in human metabolism and has been implicated with several acute and chronic effects to living organisms including humans. According to the Agency for Toxic Substances and Disease Registry (2014), some of the organ systems affected by lead, arsenic, and mercury include cardiovascular, gastrointestinal, neurological, renal, reproductive, and respiratory. It is gonadotoxic and embryotoxic as a result of its ability to cross the placental barrier (Hu 2002; Galadima and Garba 2012; Inoue 2013). In similar vein, cadmium is known to bioaccumulate in the kidney for a relatively long time, ranging from two to three decades, and at high doses, is also known to produce health effects on the respiratory system and induce the renal and hepatic toxicity and has been associated with bone disease, poor reproductive capacity, hypertensions, tumors, and hepatic dysfunction (Waalkes 2000). Although, beneficial to plants and animals in minute quantity, chromium and nickel become toxic in high concentration and can cause gastrointestinal hemorrhage, hemolysis, acute renal failure, chest pain, encephalopathy, reduced sperm count, pulmonary fibrosis, and lung cancer (Inoue 2013; Suzuki et al. 2017).

In recent years, polycyclic aromatic hydrocarbons (PAHs) have been detected in the environment in a worrisome proportion and this poses great risk to food crops as most PAHs including benzo[a]pyrene, benzo[k]fluoranthene, dibenzo[a,h]anthracene, benzo[g,h,i]perylene, and indeno[123-cd]pyrene are highly lipid soluble and thus can bioaccumulate in tissues of both plants and animals (WHO 2000; Gehle 2009; Wick et al. 2011). PAHs undergo metabolic activation in mammalian cells to diol epoxides that bind covalently to cellular macromolecules, such as DNA, RNA, and proteins, resulting in cell transformation, thereby causing errors in DNA replication and mutations that initiate the carcinogenic process (Eisler 1987; Mafra et al. 2010). Indeed, heavy metals and PAHs could result in disastrous outcome in humans if their levels are above the permissible levels in food such as instant noodles.

From the foregoing, it could be potent to assert that the occurrence of heavy metals and PAHs in instant noodles can be dangerous to humans. However, there is a need to estimate the potential risk involved in the consumption of these noodles as this approach has overtime proven to be effective in assessing the impact of hazards to human health. There are several methods for estimating potential risks of heavy-metal intake in noodles to human health (Zhang et al. 2012). The risks may be carcinogenic or non-carcinogenic (Yi et al. 2011; Sultana et al. 2017). At present, non-cancer risk assessment methods do not provide quantitative estimate of the probability of experiencing non-cancer effects from contaminant exposure. Thus, these methods are typically based on the Target Hazard Quotients (THQ).

Although the THQ-based risk assessment method does not provide a quantitative estimate of the probability of an exposed population experiencing an adverse health effect, it does provide an indication of the risk level associated with pollutant exposure over a specified period of time (Bempah and Ewusi 2016). This method of risk estimation has been shown to be valid and useful, by many researchers (Chien et al. 2002; Wang et al. 2005). As the consumption of instant noodles is growing at an astronomical rate in Nigeria and indeed the world as evidenced in the consumption of over 100 billion packs of noodles worldwide in 2012, with China constituting 44% of global demand (Tolaram Group 2012; IMARC Group 2017), there is need for intensive monitoring of ready meals and convenience food for consumer safety. Hence, this study seeks to assess the potential health risks associated with the consumption of selected instant noodle brands commonly sold in Port Harcourt, Nigeria.

## Materials and methods

### Sampling

According to reports by Marketing Edge (2017), of the 16 competing instant noodle brands in the market, six are currently in the high-growth market and account for over 70% share of noodles sold in all regions of Nigeria. Thus, samples of these six commercial branded noodles were purchased from retail shops in Choba, Alakahia, Rumuosi, Rumualogu, Ozuoba, and Rumuokoro, all within Port Harcourt Metropolis, Rivers State, Nigeria.

### Sample preparation

Noodle samples were taken randomly from the composite sample and dried at 105 °C for 24 h for thorough drying, after which it was ground to powder form using mortar. The ground solid samples (5 g each) were placed in crucibles and few drops of concentrated nitric acid were added to the solid as an ashing aid. The process was carried out in a muffle furnace by stepwise increase of the temperature up to 550 °C and then left to ash at this temperature for 4 h (Crosby 1977). The ash was left to cool and then rinsed with 1 M nitric acid. The ash suspension was filtered and the filtrate made up to the volume of 25 ml with 1 M nitric acid for metal analysis using atomic absorption spectrophotometry.

### Spectroscopic analysis

Atomic absorption spectrophotometer model Varian AA 280 was used for the spectroscopic analysis for Pb, As, Ni, Hg, Cu, Cd, Al, and Cr (AOAC 1998). The sample solutions were subsequently analyzed for heavy metal contents, on dry-weight basis. For the determination, three solutions were prepared for each sample and three separate readings were made for each solution. The means ± SD of these figures were used to calculate the concentrations.

### Determination of polycyclic aromatic hydrocarbons levels

Samples were extracted with dichloromethane and subjected to gas chromatographic analysis. Extraction was carried out according to TNRCC TX method 1005 (1997).

### Human health risk assessment

In evaluating the level of human exposure to heavy metals in contaminated noodles, the recommendations of the United States Environmental Protection Agency (US EPA 1989; 1992; 2003; 2005) were adopted and the estimated average daily intake (ADI_e_) (mg/kg/day) for adults was determined by the following equation:

$$ \kern5.75em \mathrm{ADIe}=\frac{\mathrm{Cm}\times \mathrm{IRn}\times \mathrm{EFh}\times \mathrm{EDf}\ }{\mathrm{Baw}\times \mathrm{ATd}} $$

where C_m_ is the metal concentration in noodle samples (mg/kg) and IRn is the noodles ingestion rate per day (kg/person/day). According to US EPA (2005), food intake for an adult is considered to be 0.310 kg/person/day. EFh is the exposure frequency (365 days/year) while EDf is the exposure duration (70 years). B_aw_ is the average body weight of exposed individual, considered to be 60 kg for an African adult (Walpole et al. 2012; FAO 2011), and ATd is the 70-year lifetime for carcinogenic effects (70 years × 365 days/year) (US EPA 2003, 2005; Turdi, M. and Yang, L. 2016).

#### Non-carcinogenic health effect (target hazard quotient) of heavy metals in the noodles

The non-carcinogenic health risks from consumption of sampled brands of noodles were assessed based on the THQ. The method of estimating risk using THQ (Chien et al. 2002; USEPA 2011) is based on the equation below:

$$ \mathrm{THQ}=\frac{\mathrm{ADIe}}{{\mathrm{RfD}}_{\mathrm{ing}}} $$

where THQ is the Target Hazard Quotient and ADIe is estimated average daily intake (mg/kg/day) while RfD_ing_ is the oral reference dose (mg/kg/day). RfD_ing_ is an estimate of a daily oral exposure for human population, which does not cause deleterious effect during a lifetime, generally used in EPA’s non-cancer health assessment. Values of RfD_ing_ (mg/kg/day) for Cr, Cd, As, Pb, Al, Hg, Ni, and Cu were taken from the Integrated Risk Information System (USEPA 2011; FAO/WHO 2011). Table 1.

**Table 1:** Toxicological characteristics of the investigated heavy metals

USEPA 2011 FAO/WHO 2011	Ingestion reference dose	Ingestion carcinogenic slope factor
	RfD_ing_(mg kg ^−1^d^−1^)	CSF_ing_(mg kg ^−1^d^−1^)^−1^
Pb	35E-4	85E-4
Cr	15E-1	5E-1
As	3E-4	15E-1
Ni	2E-2	17E1
Cd	1E-3	38E-2
Hg	1E-4	0
Al	70E-1	–
Cu	4E-2	–

### Hazard index

The risk assessment of a mixture of heavy metals and the individual HQs of each heavy metal are combined to form the hazard index (HI), where an HI > 1 means an unacceptable risk of non-carcinogenic effects on health, while an HI < 1 means an acceptable level of risk (ECETOC 2001). The modification is appropriate!

### Carcinogenic health effect (carcinogenic risk) of heavy metals in the noodles

Lifetime probability of contracting cancer due to exposure to chemicals is calculated as follows:

$$ \mathrm{Lifetimeprobabilityofcancer},\mathrm{carcinogenicrisk}=\mathrm{ADIe}\times {\mathrm{CSF}}_{\mathrm{ing}} $$

where CSF_ing_ is ingestion cancer slope factor (mg/kg/day)^−1^.

In 2011, the USEPA proposed 10^−6^ (1 in 1,000,000) to 10^−4^ (1 in 10,000) a permissible range for carcinogenic risk over a lifetime of 70 years (USEPA 2011).

## Statistical analysis

Unless otherwise indicated, all measurements were performed in triplicates and on at least two different occasions. Statistical analysis was done using Statistical Package for the Social Sciences (SPSS Inc., Chicago, IL, USA. Version 20).

### Results

The assessment of heavy metal content in six brands of instant noodles revealed that the noodles had varying concentrations of heavy metals (Table 2). The concentrations of Pb in samples of six noodle brands ranged from 0.043 ± 0.15 to 3.163 ± 0.21 mg/kg with highest average (mean ± SD) concentrations observed in CFN (3.163 ± 0.21 mg/kg) and GFC (1.022 ± 0.08 mg/kg) which were significantly higher (*P* ≤ 0.05) than those in NGP (0.043 ± 0.15 mg/kg) and GAA (0.276 ± 0.18 mg/kg), although all were above WHO permissible limit for Pb in food (WHO 2011). However, Pb was not detected in brands CUN and FCS. Similarly, the concentration of Cu in noodle brands ranged between 0.021 ± 0.01 and 0.119 ± 0.10 mg/kg, with CFN recording the highest average (mean ± SD) concentration (0.119 ± 0.10 mg/kg), significantly higher (*P* ≤ 0.05) than observed Cu concentrations in other brands. Ni was detected in three (GAA, CUN, and FCS) of the six brands analyzed with concentrations ranging from 0.012 ± 0.01 to 0.354 ± 0.20 mg/kg, marginally different from one another but all below the WHO standard for Ni in food (WHO 2011), while concentration of Al, on the other hand, was between 0.014 ± 0.11 and 0.237 ± 0.11 mg/kg, all within permissible standard, with NGP and FCS recording the highest and lowest average (mean ± SD) concentrations (0.237 ± 0.11 and 0.014 ± 0.11 mg/kg), respectively. Cr concentration in samples of noodle brands ranged from 0.041 ± 0.11 to 2.421 ± 0.71 mg/kg with samples GAA recording highest average (mean ± SD) concentration (2.421 0.71 mg/kg), significantly higher (*P* ≤ 0.05) than those of other brands [CFN (0.504 ± 0.09), NGP (0.041 ± 0.11), and FCS (0.159 ± 0.11) mg/kg] and above permissible standard for Cr (0.050 mg/kg). Interestingly, As, Hg, and Cd were not detected in samples of the six noodle brands analyzed.

**Table 2:** Results of heavy metal analysis (mg/kg) in selected noodle samples (mean ± SD, *n* = 3)

Heavy metals	CFN	GFC	NGP	GAA	CUN	FCS	WHO standard
Pb	3.163 ± 0.21*	1.022 ± 0.08*	0.043 ± 0.15	0.276 ± 0.18	ND	ND	0.025
As	ND	ND	ND	ND	ND	ND	0.015
Ni	ND	ND	ND	0.012 ± 0.01	0.075 ± 0.17	0.354 ± 0.20	1.00
Hg	ND	ND	ND	ND	ND	ND	0.005
Cu	0.119 ± 0.10*	0.068 ± 0.16	0.038 ± 0.01	0.044 ± 0.03	0.027 ± 0.09	0.021 ± 0.01	NA
Cd	ND	ND	ND	ND	ND	ND	0.003
Al	0.134 ± 0.10	0.019 ± 0.11	0.237 ± 0.11	0.015 ± 0.18	0.027 ± 0.12	0.014 ± 0.11	0.237
Cr	0.504 ± 0.09	ND	0.041 ± 0.11	2.421 ± 0.71*	ND	0.159 ± 0.11	0.050

*ND* not detected, *NA* not available

*Significantly different at *P* ≤ 0.05

The ADIe, THQ, HI, and carcinogenic risk were used to assess the human health risk associated with trace metal contamination in samples of the six brands of noodles (Tables 3, 4, and 5). THQ was in the order Pb > Hg > As > Cu > Cd > Cr > Ni > Al and Pb > Hg > As > Cu > Ni > Cd > Al > Cr in samples of brands CFN and GFC, respectively. Notably, THQ values for Pb contamination in brands CFN and GFC (4.91 and 1.59, respectively) were greater than 1 (THQ > 1) while values were less than 1 (THQ ˂ 1) for all other metals in all six noodle brands considered. Similarly, HI values for brands CFN and GFC (5.004 and 1.680, respectively) were greater than 1 while brands NGA, GAA, CUN, and FCS had values less than 1 (HI ˂ 1).

**Table 3:** Estimated average daily intake (ADIe) and target hazard quotient (THQ) of heavy metals (mg/kg/day) through consumption of noodles in Port Harcourt, Nigeria

Samples		Pb	As	Ni	Hg	Cu	Cd	Al	Cr
CFN	ADIe THQ	17.2E-3 4.91*	5E-6 18E-3	5E-6 3E-4	5E-6 54E-3	6E-4 16E-3	5E-6 5E-3	7E-4 3E-4	3E-4 6E-4
GFC	ADIe THQ	56E-4 1.59*	5E-6 18E-3	5E-6 3E-4	5E-6 54E-3	4E-4 9E-3	5E-6 5E-3	1E-4 1E-4	5E-6 4E-6
NGP	ADIe THQ	2E-4 67E-3	5E-6 18E-3	5E-6 3E-4	5E-6 54E-3	2E-4 5E-3	5E-6 5E-3	13E-4 1E-3	2E-4 1E-4
GAA	ADIe THQ	15E-4 42.9E-2	5E-6 18E-3	1E-4 3E-3	5E-6 54E-3	2E-4 6E-3	5E-6 5E-3	1E-4 3E-5	13.2E-3 9E-3
CUN	ADIe THQ	5E-6 2E-3	5E-6 18E-3	4E-4 21E-3	5E-6 54E-3	1E-4 4E-3	5E-6 5E-3	1E-4 1E-4	5E-6 4E-6
FCS	ADIe THQ	5E-6 2E-3	5E-6 18E-3	2E-4 96E-3	5E-6 54E-3	1E-4 3E-3	5E-6 5E-3	1E-4 1E-4	8E-4 6E-4

*Indication of potential health risk (THQ ≥ 1)

**Table 4:** Hazard index of heavy metals through consumption of noodles in Port Harcourt

Samples	CFN	GFC	NGP	GAA	CUN	FCS
Hazard Index	5.004*	1.680*	0.150	0.135	0.104	0.179

*Indication of potential health hazard (HI ≥ 1)

**Table 5:** Carcinogenic risks (CR) of heavy metals through consumption of noodles in Port Harcourt

Samples	Pb	As	Ni	Cd	Cr
CFN	15E-4	81E-6	92E-6	21E-6	15E-4
GFC	48E-6	81E-6	92E-6	21E-6	27E-6
NGP	20E-6	81E-6	92E-6	21E-6	10E-4
GAA	13E-5	81E-6	17E-4	21E-6	66E-3*
CUN	46E-8	81E-6	68E-4	21E-6	27E-6
FCS	46E-8	81E-6	32E-4	21E-6	40E-4

*Value higher than permissible range

The level of PAHs in the noodle samples as shown in Table 6 has total PAHs ranging from 564 ± 0.61 to 7.887 ± 0.73 mg/kg with brands CFN and CUN having average (mean ± SD) total PAH contents (7.887 ± 0.73 and 7.457 ± 0.51 mg/kg, respectively) significantly higher (*P* ≤ 0.05) than values observed in samples of other brands (GAA, NGP, and GFC) even though values for GAA (5.549 ± 0.69 mg/kg) and NGP (5.218 ± 0.47 mg/kg) were higher compared to GFC (0.564 ± 0.61 mg/kg). On the other hand, total carcinogenic PAHs in the noodle brands ranged between 0.564 ± 0.61 and 5.615 ± 0.93 mg/kg. Average contents (mean ± SD) of carcinogenic PAHs in brands CUN and CFN (5.615 ± 0.93 and 5.521 ± 0.49 mg/kg, respectively) were significantly higher (*P* ≤ 0.05) than those observed for brands GAA (4.640 ± 0.47 mg/kg), FCS (2.804 ± 0.54 mg/kg), NGP (2.727 ± 0.18), and GFC (0.564 ± 0.61 mg/kg).

**Table 6:** Results of PAHs (mg/kg) of selected noodle brands commonly consumed in Port Harcourt (mean ± SD, *n* = 3)

PAHs	CFN	GFC	NGP	GAA	CUN	FCS
Naphthalene	–	–	–	–	–	–
Acenaphthylene	–	–	–	0.243 ± 0.35	–	–
2-Bromo-naphthalene	0.700 ± 0.19	–	1.410 ± 0.83	–	0.231 ± 0.12	0.259 ± 0.47
Acenaphthene	–	–	–	–	–	–
Fluorene	0.660 ± 0.71	–	0.170 ± 0.27	0.124 ± 0.92	0.340 ± 0.74	0.204 ± 0.39
Phenanthrene	1.008 ± 0.95	–	0.911 ± 0.46	0.463 ± 0.36	0.906 ± 0.29	0.601 ± 0.82
Anthracene	–	–	–	0.084 ± 0.55	0.079 ± 0.31	–
Fluoranthene	–	–	–	–	0.155 ± 0.43	–
Pyrene	–	–	–	–	0.131 ± 0.68	–
Benzo[a]anthracene^**a**^	0.655 ± 0.30	–	1.270 ± 0.59	1.760 ± 0.72	0.387 ± 0.50	0.557 ± 0.45
Chrysene^**a**^	0.409 ± 0.53	–	0.871 ± 0.14	0.808 ± 0.49	0.649 ± 0.72	0.287 ± 0.16
Benzo[b,j,k]fluoranthene^**a**^	0.464 ± 0.47	–	0.229 ± 0.64	1.370 ± 0.32	0.200 ± 0.19	1.490 ± 0.24
Benzo[a]pyrene^**a**^	0.834 ± 1.05	0.564 ± 0.61	0.357 ± 0.58	0.702 ± 0.81	0.660 ± 0.23	0.470 ± 0.53
Indeno[1,2,3-c,d]pyrene^**a**^	1.166 ± 0.14	–	–	–	2.549 ± 0.93	–
Dibenzo[a,h]anthracene^**a**^	1.280 ± 0.69	–	–	–	1.170 ± 0.47	–
Benzo[g,h,i]perylene^**a**^	0.713 ± 0.85	–	–	–	–	–
Total PAHs	7.889 ± 0.73*	0.564 ± 0.61	5.218 ± 0.47	5.554 ± 0.69	7.457 ± 0.51*	3.868 ± 0.80
**∑** of carcinogenic PAHs	5.521 ± 0.49*	0.564 ± 0.61	2.727 ± 0.18	4.640 ± 0.47	5.615 ± 0.93*	2.804 ± 0.54
% carcinogenic PAHs	69.980	100.0	52.260	83.540	75.298	72.492

^a^Carcinogenic PAHs

*Significantly different at *P* ≤ 0.05

## Discussion

Instant noodles are a household food common among students and children in Nigeria, and its consumption has continued to grow on a daily basis because of the convenient method of preparation. Recently, the public and stakeholders, especially non-governmental organizations have raised concerns about the safety of noodles supplied to consumers in Port Harcourt, Nigeria, as they made public their reservation about the processes employed in the production of these noodles. Heavy metals are the commonest contaminants that could be detected in food as they can contaminate food through the raw materials or equipment used in the production process (Li et al. 2014). The current study demonstrates that heavy metals and PAHs are present in some of the noodles sold in Port Harcourt metropolis and assesses the potential health risks associated with their consumption.

The assessment of heavy metals in the samples showed that Pb, Cr, Cu, Ni, and Al are present in the noodles in varying concentrations (Table 2). The levels of Pb in samples CFN, GFC, NGP, and GAA are above the World Health Organization (WHO 2011) permissible limits (0.025 mg/kg) and thus could have adverse effects on health especially the central nervous system, the cardiovascular system, the kidneys, and the immune system (Bergeson 2008; Rossi 2008); however, Pb was not detected in samples CUN and FCS, an indication that both noodles are not contaminated by Pb. The contamination may have been from raw ingredients such as wheat flour used in noodle production. In their study, Ali and Al-Qahtani (2012) observed that high concentrations of heavy metals in food might be related to their concentrations in the polluted air with industrial activities. Oil exploration and exploitation activities have been taking place in the Niger Delta for about five decades (Emuedo et al. 2014; Oviasuyi and Uwadiae 2010) and it might be reasonable to speculate that industrial activities could be a major source of Pb in the environment (Maleki and Zarasvand 2008; Amnesty International 2009). Also, Pb contamination could arise from irrigation of wheat farmland with contaminated water, application of fertilizer and metal-based pesticides, industrial emissions, and transportation as well as the method of harvesting and storage. Similar work on instant noodles by Satsananan (2017) showed Pb was within the WHO set limit.

The concentration of Cr in samples CFN, NGP, GAA, and FCS exceeds the limit set by the WHO (0.050 mg/kg). Chromium (as Cr^3+^) is an essential dietary element and occurs naturally in many vegetables, fruits, meats, and grains; thus, it is beneficial to health in that form; however, Cr^6+^ at high levels can cause gastrointestinal hemorrhage, hemolysis, acute renal failure, reduced sperm count, pulmonary fibrosis, and lung cancer (Inoue 2013; Suzuki et al., 2017). Although beneficial to health, in vitro studies indicated that high concentrations of Cr (III) in the cell can lead to DNA damage (Eastmond et al. 2008; Trichopoulos et al. 1997). Like Pb, Cr contamination in the noodles might be due to industrial and unsafe agricultural practices. Nickel was detected in the brands coded GAA, CUN, and FCS; however, it is below the maximum permissible limit (1 mg/kg). Thus, it may pose no Ni-based toxicity to consumers (BSTI 2001). Similarly, the levels of Al are within the acceptable standards of 0.014 and 0.237 mg/kg, respectively (WHO 2003; BSTI 2001), whereas As, Hg, and Cd were not detected in any of the noodle samples studied.

The human health risk assessment of the heavy metals showed low target hazard quotient (THQ) for most of the noodles, suggesting an acceptable level of non-carcinogenic adverse risk (Table 3). However, unacceptable THQ was observed for Pb in samples CFN and GFC. Thus, continuous intake of both noodles is likely to induce deleterious health risk arising from the Pb contamination. However, in reality, the ingested dose of heavy metal is not equal to the absorbed pollutant because a fraction of the ingested heavy metals may be excreted while the remnant can bioaccumulate in body tissues where they affect human health adversely (Zhuang et al. 2009). Accordingly, the health hazard index (HI) for samples CFN and GFC are > 1 suggesting the unacceptable risk of non-carcinogenic effects on health (Table 4). Heavy metals have the potential to bioaccumulate and thus an insignificant level of a particular heavy metal can bioaccumulate in consort with other metals and pose great risk to human health (Tao et al. 2012). From the foregoing, it could be safe to assert that samples CFN and GFC are unsafe for human consumption as the THQ and HI are > 1 and the level of Pb in both samples is above the WHO permissible limit. A brief look at the carcinogenic risk revealed that all noodle samples had values within the permissible range published by USEPA, except for Cr in sample GAA (U.S. EPA, 2011).

Polycyclic aromatic hydrocarbons (PAHs) are ubiquitous and their occurrence in food, water, soil, and the atmosphere is mainly of anthropogenic origin (Alberta Air Research Users Group 2000), and their detection in the noodle samples (Table 6) is an indication that industrial activities could be the major contributor. The production of PAHs in industries has been reported to be favored by an oxygen-deficient flame, temperatures in the range of 650–900 °C and fuels which are not highly oxidized (Maliszewska-Kordybach 1999). The high level of total PAHs in sample CFN is worrisome as a bulk of it are carcinogenic PAHs (benzo[a]pyrene, benzo[b,j,k]fluoranthene, dibenzo[a,h]anthracene, benzo[g,h,i]perylene, and indeno[123-cd]pyrene). This noodle (CFN) might not be safe for consumption because PAHs are highly lipid soluble and can bioaccumulate in tissues of both plants and animals (Gehle 2009; Wick et al. 2011). More so, long-term occupational exposures to PAHs mixtures have been associated with increased incidence of lung, skin, stomach, gastrointestinal tract, bladder, and scrotal cancer (Government of South Australia 2009). Similarly, the detection of PAHs, especially carcinogenic PAHs in CUN and GAA, could pose serious health risk, in the long term, to consumers.

## Conclusion

This study assessed the human health risk from heavy metals and PAHs in six instant noodles sold in Port Harcourt, Nigeria. The levels of heavy metals (especially Pb and Cr) in some brands of the noodles (CFN, GFC, NGP, and GAA) were above the maximum permissible limits established by WHO. The continuous consumption of these noodles may lead to heavy metal toxicity which could result in impaired neuronal and renal functions. Although some of the heavy metals are within the permissible limit, their consumption could still be unsafe because of the cumulative potential of these contaminants. Moreover, the health risk assessment further confirmed the propensity for toxicity to occur if samples CFN and GFC are consumed; two brands with remarkably high THQ and HI. On the other hand, consumers of brands CUN, CFN, and GAA may be predisposed to carcinogenesis due to the high PAH content in the brands. Hence, consumption of these brands should be greatly minimized, if not completely avoided.

It is strongly recommended that regulatory agencies of government such as the National Agency for Food, Drug Administration and Control, Standards Organization of Nigeria, and Consumer Protection Council should strictly monitor the activities of these noodles producers to ensure only wholesome noodles are supplied to consumers.

## Abbreviations

WHO: World Health Organization
NAFDAC: National Agency for Food and Drug Administration and Control
CPC: Consumer Protection Council
SON: Standards Organization of Nigeria
